# An Interesting Operation

**Published:** 1872-05

**Authors:** Mary J. Safford

**Affiliations:** Chicago.


### ARTICLE XV.

### *An Interesting Operation.*

FROM THE GERMAN. BY MARY J. SAFFORD, M. D., OF CHICAGO.

From Munich comes a report of the following operation, made Feb. 15, 1872, by the Royal Bavarian physician, Prof. Nurzbaum:

Soldier H., aged 24, in the engagement at Bazeilles, received a blow upon the elbow and nape of the neck with the butt end of a needle gun, in consequence of which he suffered continually with spasms, from a tonic contraction of the muscles, so that at times he became unconscious. Different remedies were used, such as vesicants, gymnastics and baths of all kinds, all in vain. After consultation with the Professor of Physiology, Dr. Virt, it was decided that the seat of the affection was in the spine—that is at the origin of the roots of the nerves—and that only such remedies would be of use which acted directly upon the spine. As the patient was accused of feighing his sufferings he was kept in confinement where Prof. N. had ample opportunity to observe him, and he decided to lay bare and to stretch all the nerves of the spine that came into action with the disease, and to follow the four lower cervical nerves as far as possible to their exit from the spine, and by distending them, to perhaps work favorably upon the neighboring spinal portion; and by freeing some adhesions to the spinal foraminæ, thus to relieve the tonic spasms. The nature of the operation was explained to the patient, who gave his consent to it.

He was completely narcotized to the successful end of the operation. By exposing and distending the four lower cervical nerves, the paralysis of the sensitive nerves and the spasms of the mater nerves was conquered.

Thus is proven the possibility, by operative means upon the spine, of removal of paralysis and of spasms.

Prof. Nurzbaum remarked in his report: "I am glad to have made the operation before a hundred witnesses, so that no jester can say I have dreamed of the operation, instead of making it."—*Medical Examiner.*

